# Ecological Burden of e-Waste in Bangladesh—an Assessment to Measure the Exposure to e-Waste and Associated Health Outcomes: Protocol for a Cross-sectional Study

**DOI:** 10.2196/38201

**Published:** 2022-08-16

**Authors:** Sarker Masud Parvez, Shaikh Sharif Hasan, Luke D Knibbs, Farjana Jahan, Mahbubur Rahman, Rubhana Raqib, Nafisa Islam, Nirupam Aich, Mohammad Moniruzzaman, Zahir Islam, Masatake Fujimura, Peter D Sly

**Affiliations:** 1 Children’s Health and Environment Program Child Health Research Centre The University of Queensland Brisbane Australia; 2 Environmental Interventions Unit Infectious Diseases Division International Centre for Diarrheal Disease Research, Bangladesh Dhaka Bangladesh; 3 School of Public Health Faculty of Medicine and Health The University of Sydney Sydney Australia; 4 Public Health Unit Sydney Local Health District, Camperdown Sydney Australia; 5 Department of Chemical Engineering Bangladesh University of Engineering and Technology Dhaka Bangladesh; 6 Department of Civil, Structural and Environmental Engineering School of Engineering and Applied Sciences University at Buffalo, The State University of New York Buffalo, NY United States; 7 Bangladesh Council of Scientific & Industrial Research Dhaka Bangladesh; 8 Department of Basic Medical Sciences National Institute for Minamata Disease Minamata City, Kumamoto Japan

**Keywords:** e-waste, lead, cadmium, mercury, environment, exposure, recycling, toxicants, health outcomes, Bangladesh

## Abstract

**Background:**

e-Waste is a rapidly growing waste stream worldwide, and Bangladesh is a hub of e-waste handling. Informal e-waste recycling operations involve crude methods for dismantling, repairing, sorting, and recycling electronic goods with bare hands and without personal health protections. Direct inhalation or dermal exposure to toxicants during informal recycling is common. Evidence suggests that e-waste–derived toxicants pollute the terrestrial ecosystem and have been linked with adverse health effects. However, e-waste recycling–related occupational health hazards have not been adequately explored in the context of Bangladesh.

**Objective:**

Our study aims to expand the current understanding of exposure to e-waste. This study will measure the metal concentrations in biological and environmental samples and evaluate the relationship between heavy metals and the biochemical systems of the e-waste workers.

**Methods:**

The study uses a cross-sectional study design consisting of an exposed site and a nonexposed control site. The trained team collected information on individual exposures, detailed work and medical history, and biological samples (blood, urine, and hair) from each subject. This study will measure heavy metal levels (lead, cadmium, and mercury) and biochemical parameters (hematological, hormonal, renal, and others) from the biological samples with reported physical function as outcomes of interest. In addition, we also collected soil and dust samples from both exposed and nonexposed control sites to measure the health risk. All the environmental samples will be analyzed using inductively coupled plasma mass spectrometer to determine metal concentrations. We will also conduct a qualitative investigation for a deeper understanding of the e-waste management system in Bangladesh.

**Results:**

The protocol has been approved by the Institutional Review Boards of the International Centre for Diarrheal Disease Research, Bangladesh, and The University of Queensland’s Human Behavioral Ethics Committee. Informed written consent was obtained from all participants. We recruited 199 workers from the e-waste sites with at least 5 years of exposure and 104 control subjects with no industrial or e-waste exposure. Sample analysis is estimated to be completed in 2022.

**Conclusions:**

Although many studies have identified potential adverse health outcomes from exposure to e-waste, there is a lack of published epidemiological research in Bangladesh. Research in this field is particularly pressing in the context of the current e-waste trend and the need to deepen the understanding of exposures and outcomes.

**International Registered Report Identifier (IRRID):**

DERR1-10.2196/38201

## Introduction

### Background

e-Waste is a rapidly growing waste stream that poses severe threats to the environment and human health [1,2]. This can be defined as any “electrical or electronic equipment, which is waste, including all components, subassemblies, and consumables, which are part of the equipment at the time the equipment becomes waste” [3]. The exponential growth of the electronic industries in the last couple of decades has resulted in a considerably high volume of obsolete waste flow [4-8]. In 2014 and 2016, the estimated quantity of e-waste was about 41.8 and 44.7 million metric tons, respectively, all over the world, whereas only about 15% (6.5 million metric tons) and 20% (8.9 million metric tons) were formally collected and recycled by a proper channelized system. In 2019, Global E-waste Monitor estimated the global production of e-waste at approximately 53.6 million metric tons. This figure is expected to grow to 74.7 million metric tons by 2030, with the majority of e-waste produced in Asia (24.9 million metric tons) [9]. Low-income countries are attractive destinations for e-waste due to lower labor costs, cheaper disposal, and less stringent or poorly enforced laws, with illegal export from high-income countries accounting for approximately 80% of the burden [10,11].

e-Waste contains large amounts of hazardous material. High concentrations of known neurotoxicants, including lead (Pb), cadmium (Cd), chromium (Cr), polybrominated diphenyl ether, polychlorinated biphenyl compounds, and polycyclic aromatic hydrocarbons are leached or discharged from e-waste [12]. Exposure to these pollutants can cause toxicity to the respiratory, circulatory, nervous, immune, endocrine, urinary, and reproductive systems [13-15]. Additionally, e-waste–derived metals are nonbiodegradable, which adversely affects aquatic and terrestrial environments [16].

e-Waste exposure usually comes from 1 of 3 sources: informal recycling, formal recycling, or environmental contamination [17-20]. Most e-waste recycling processes are carried out in the informal sector, where the recycling process is used to extract valuable metals rapidly. These crude operations are carried out without protective gear or the assistance of technology [21,22]. Workers who handle e-waste as part of their formal or informal occupations are more likely to be exposed through inhalation, ingestion, and dermal absorption, and this is typically known as direct occupational exposure [23-25]. In addition, local inhabitants and workers can also be exposed through indirect routes, including physical contact with contaminated soil, dust, air, water, and food sources [26,27]. Several studies reported that even if the residents live nearby the recycling areas, they are still at risk of exposure due to the high load of contamination [25,28].

### Country Context and Rationale of the Study

Bangladesh has a rapidly growing economy, which is parallel with the proliferation of e-waste from electronic gadgets, especially mobile phones and computers [29]. The Bangladeshi Department of Environment estimated that 0.40 million metric tons of e-waste were generated in 2018, and this is expected to reach 4.62 million metric tons by 2035 with an annual growth rate of around 20% [30]. However, only 3% of e-waste enters the market of recycling, and the rest is mixed with municipal solid waste and goes to the landfill [30]. Recently, Hazardous Waste (e-waste) Management Rules, 2021 under the Bangladesh Environment Conservation Act, 1995 were approved by the Government of Bangladesh [31]. This initiative has the potential to markedly improve the management of e-waste and reduce the harm to human and environmental health. However, extensive research is required to identify and develop effective policies. Our study aims to fill the gaps in the current understanding of how e-waste handling contributes to adverse consequences on human and environmental health.

### Study Objectives

**Figure 1:**
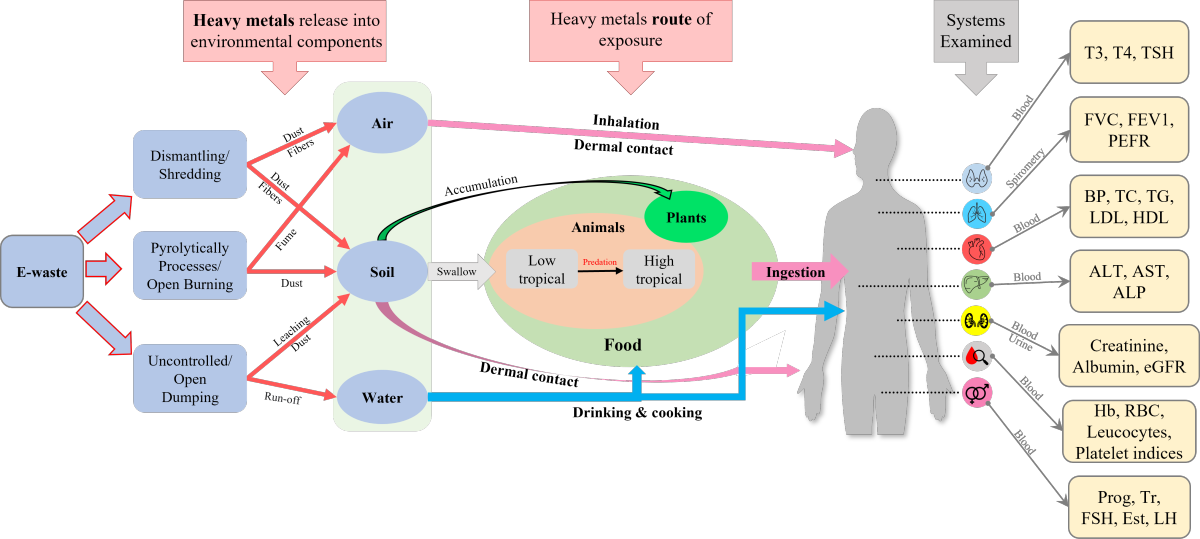
Study conceptual model of the effects of exposure to e-waste on environmental and human health. ALP: alkaline phosphatase; ALT: alanine aminotransferase; AST: aspartate transaminase; BP: blood pressure; eGFR: estimated glomerular filtration rate; Est: estrogen; FEV_1_: forced expiratory volume in 1 second; FSH: follicle-stimulating hormone; FVC: forced vital capacity; Hb: hemoglobin; HDL: high-density lipoprotein; LDL: low-density lipoprotein; LH: luteinizing hormone; PEFR: peak expiratory flow rate; Prog: progesterone; RBC: red blood cell; T_3_: triiodothyronine, T_4_: thyroxine; TC: total cholesterol; TG: triglyceride; Tr: testosterone; TSH: thyroid-stimulating hormone.

The overarching objective of the study is to improve the current understanding of exposure to e-waste and its adverse consequences. Based on the literature review, we conceptualized a framework for the effects of exposure to e-waste on environmental and human health (Figure 1). We identified hot spots likely to have higher exposures within Dhaka city and the factors that determine the location of hot spots. The specific aims of the project are to (1) explore the management pathway of e-waste from collection to disposal; (2) determine the different types of e-waste that are modified physically and chemically in Dhaka city; (3) measure the frequency of Pb, Cd, mercury (Hg) and other elements (arsenic, Cr, copper, manganese, nickel, zinc, cobalt, selenium, beryllium, and vanadium) contamination of soil and dust in e-waste repair and recycling areas; 4) measure the levels of Pb and Cd in blood and Hg in hair among children, adolescents, and adults working in e-waste repair and recycling area; (5) investigate the characteristics (work exposure, behavioral, and occupational safety) associated with blood Pb and Cd and hair Hg levels among participating respondents; and (6) measure the association between heavy metals in biological samples and biochemical parameters (hematological, renal, hormonal, respiratory, cardiovascular, and liver function and oxidative damage markers) of the e-waste workers. All of these objectives will be assessed by comparing e-waste workers to those living in unexposed areas.

## Methods

### Study Design

**Figure 2:**
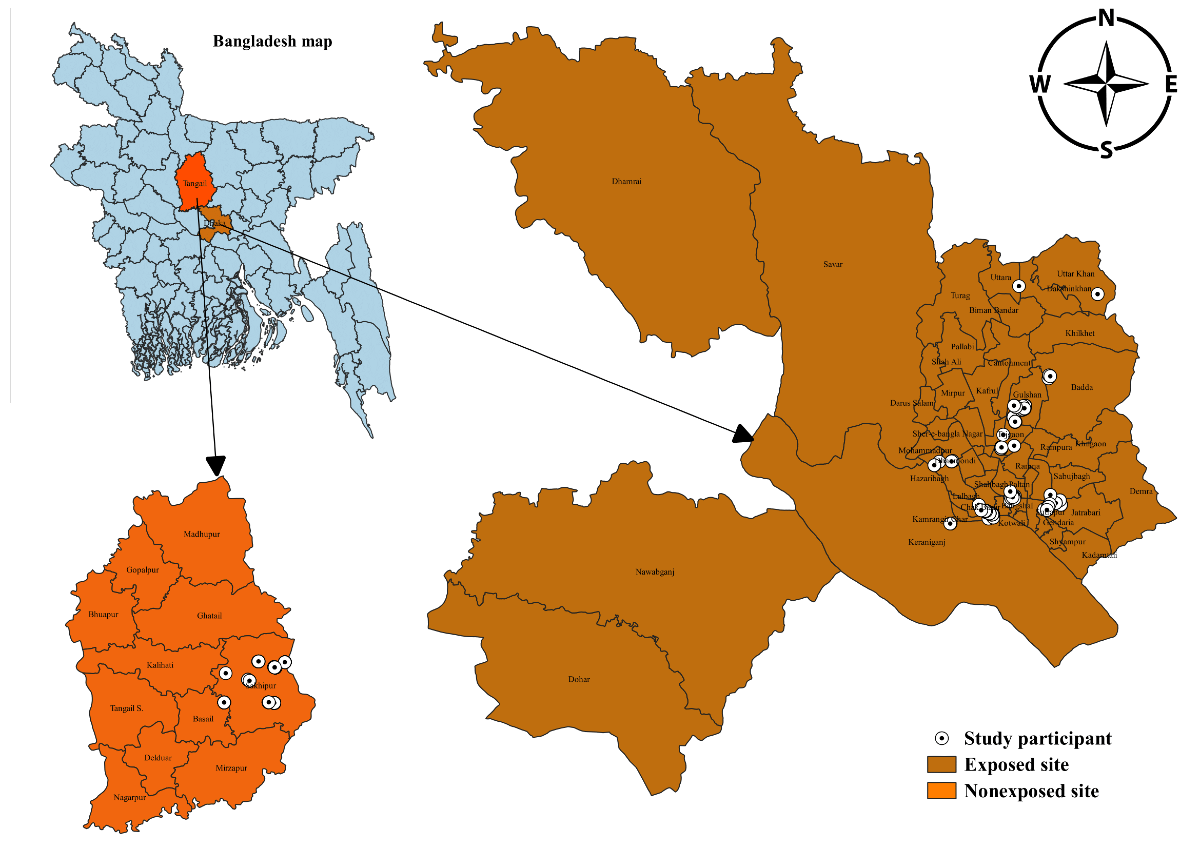
Study sites.

The design comprises both quantitative and qualitative investigations to meet the objectives. Our emphasis is on the quantitative investigation where we use a cross-sectional study consisting of an exposed site and a nonexposed control site (Figure 2). For the in-depth understanding of e-waste management, we will also conduct a qualitative investigation.

### Study Site, Population, and Participant Recruitment

**Figure 3:**
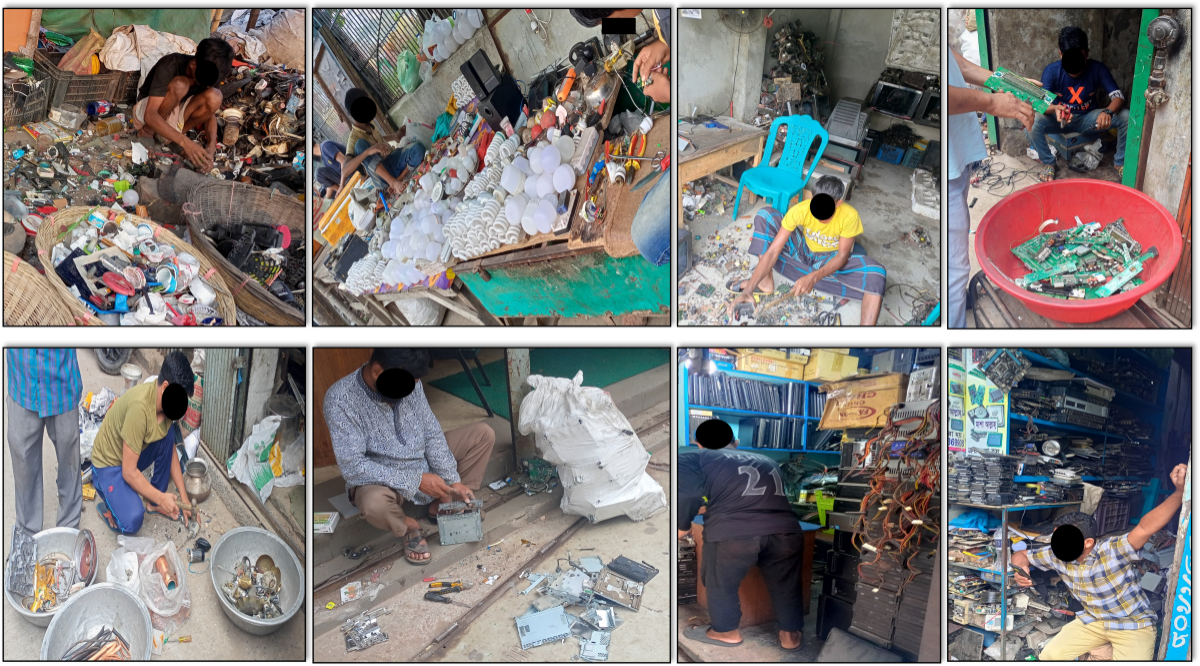
e-Waste exposure sites and working condition.

The research team identified key informal e-waste recycling sites in Dhaka (Figure 2). The study population included e-waste workers involved in collecting, recycling, repairing, incineration, and other relevant work in these informal e-waste recycling sites (Figure 3). We screened the potential participants to confirm that they fall into one of the recycling activities targeted in our study along with at least 5 years of exposure. We excluded the workers who have had shorter durations of exposure, because chemicals derived from e-waste are potentially toxic and cause adverse health outcomes from chronic exposure. We also selected a nonexposed control site at a distance from Dhaka, where no industrial or e-waste pollution sources existed. All the nonexposed participants were local residents mainly involved in agriculture, small businesses, and teaching, and none of them had previous exposure to e-waste (Figure 2).

The trained fieldworkers travelled to the eligible areas and asked the leaders or owners for permission to conduct research within their community. The research team briefly explained the study objectives, procedures, and possible measurements that we were interested in. After providing time for discussion among the workers and owners and obtaining verbal interest to participate, a field team member recorded the GPS coordinates at the location of e-waste sites and developed a line list of workers involved in e-waste processing. Later, the field team sought informed consent from the eligible workers during enrollment. The GPS coordinates were compiled and analyzed using QGIS mapping software (version 3.16.1; Open Source Geospatial Foundation). 

### Sample Size Calculation

The primary outcome of this study is blood Pb and Cd levels among the e-waste exposed and nonexposed individuals. We calculated the sample size using the mean blood Pb and Cd concentration reported by Sirichai et al [32]. They reported mean blood Pb levels of 6.61 (SD 3.07) µg/dL and 2.73 (SD 0.49) µg/dL among Thai exposed and nonexposed controls, respectively. In contrast, the mean blood Cd levels of e-waste and non–e-waste workers were 1.00 (SD 0.33) µg/L and 1.17 (SD 0.39) µg/L, respectively. The estimated sample sizes for a 2-sample means test are 18 (9 per group) and 192 (96 per group) for Pb and Cd, respectively, considering 90% of power and a 95% CI. To meet the study objective, 96 respondents from each group would be required to detect differences between exposed and unexposed individuals. However, there is a paucity of available information on the e-waste–exposed population and the harmful effects from e-waste–derived toxicants in this context. To generate rigorous evidence and to allow for the examination of interactions and mediation pathways, particularly on the e-waste–exposed population, we enrolled 199 exposed and 104 unexposed participants.

### Data Collection Procedures

Trained fieldworkers visited the site and approached the eligible participants. The fieldworkers explained the study in detail and obtained written consent from those who agreed to participate.

#### Exposure Assessment

A data collection tool was developed and piloted prior to being administered in the field. All questionnaires were administered by trained research staff and collected electronically. The team collected data in several domains:

- general characteristics (ie, age, sex, income, education, and dwelling environment),
- detailed exposure history (years of involvement, daily/weekly work time, and work type),
- occupational safety equipment, and knowledge and attitudes about harmful effects from exposure to e-waste,
- lifestyle and behaviors, including cigarette smoking, alcohol consumption, and medication use, and
- self-reported disease status, including hypertension, diabetes mellitus, chronic kidney diseases, hepatic diseases, and others.

All interviews were conducted in the workplace with direct observation supporting results.

#### Biological Sample Collection and Storage

A trained phlebotomist collected venous blood samples (7.5 mL) from the participants using trace metal–free certified needles and tubes. Our team also collected spot urine samples in a 50 mL falcon tube; all samples were transported to the laboratory in a cooler box, maintaining adequate temperature (2°C to 8°C) within 4-6 hours of collection. A medical technologist separated 2 mL of whole blood samples for heavy metal analysis, and 1 mL of blood was immediately transferred to the laboratory to measure hematological variables. Whole blood was spun (894 G relative centrifugal force) for 15 minutes at ambient temperature for plasma separation. Aliquots of packed blood and plasma were stored at –80°C at the International Centre for Diarrheal Disease Research, Bangladesh (icddr,b). Blood glucose was measured using a rapid field test kit during blood collection. All analyses, as outlined in Table 1, on stored blood and urine analyses will be performed at the Immunobiology, Nutrition, and Toxicology Laboratory of icddr,b. In addition, the trained team also collected 20 strands of hair about 10 cm in length from the scalp to measure Hg exposure. To store hair samples, we used 1 envelope for each participant. We will send the hair samples to the National Institute for Minamata disease study laboratory in Japan, where they will use oxygen combustion–gold amalgamation procedure using a Mercury Analyzer (MA2000; Nippon Instruments) to analyze mercury content in hair [33].

#### Outcome Assessment

Our primary study outcome is blood Pb and Cd concentrations. Secondary outcomes include the impacts of heavy metals on body function. In our recent published systematic review [34], we have identified the reported health consequences of e-waste exposure (Table 1).

**Table 1:** Study outcome indicators and measurement tools.

Outcome, measurement type or sample analyzed		Indicator	Measurement tool
**Heavy metal detection**			
	Blood	Pb^a^ and Cd^b^	Graphite furnace atomic absorption spectrometry
	Hair	Total Hg^c^	Oxygen combustion–gold amalgamation procedure using Mercury Analyzer (MA2000; Nippon Instruments)
**Hematological system**			
	Blood	Hb^d^, RBC^e^, leukocytes, and platelet indices	Automated 5-part (Diff.26 parameter) hematology analyzer (XS-800i; Sysmex Corporation)
**Respiratory System**			
	Lung function	FVC^f^, FEV_1_^g^, and PEF^h^	Handheld Spirometer (SP-10; Contec)
	Respiratory health	Cough, phlegm, wheeze, shortness of breath, chest illness, and family history	Modified Medical Research Council Respiratory Questionnaire [35]
**Renal system**			
	Urinary system assessment	Difficulty emptying bladder, blood or pus in urine, and micturition problem	Self-reported questionnaire
	Urine	Creatinine, albumin, and ACR^i^	Enzymatic method on analyzer (cobas c311; Roche)
	Blood	Creatinine and eGFR^j^	Semiauto electrolyte analyzer
**Cardiovascular system**			
	Blood	TC^k^, TG^l^, LDL^m^, and HDL^n^	5-part differential semiautomated hematology analyzer (XS-800i; Sysmex)
	Blood Pressure	Systolic and diastolic pressure	Digital blood pressure monitor (HEM-907; Omron)
Anthropometry		Height, weight, waist circumference, and hip circumference	Adult portable height-length measuring board (accuracy: 0.1 cm; ShorrBoard) and Body composition monitor (HBF-214; Omron)
**Thyroid function**			
	Blood	T_3_^o^, T_4_^p^, and TSH^q^	Electrochemiluminescence immunoassay with automated immunoassay analyzers (cobas e601; Roche)
**Liver function**			
	Blood	ALT^r^, AST^s^, and ALP^t^	Semiautomatic biochemistry analyzer (Evolution 3000; BSI)
**Reproductive system**			
	Blood	Progesterone, testosterone, follicle-stimulating hormone, estrogen, and luteinizing hormone	Electrochemiluminescence immunoassay with automated immunoassay analyzers (cobas e601; Roche)
	Reproductive health assessment for women	Pregnancy history, abortion, stillbirth, menstruation history, and delivery complications	Modified version of the Core Questionnaire from the 2005 World Health Organization Multi-country Study on Women’s Health and Domestic Violence Against Women [36] and the reproductive health assessment toolkit from Centers for Disease Control and Prevention [37].
**Pro-inflammatory cytokines**			
	Blood	IL-1β^u^, IL-6^v^, IL-8^w^, IP-10^x^, IL-12_P_70^y^, and TNF-α^z^	Enzyme-linked immunosorbent assay
**Oxidative stress**			
	Urine	8-OHdG^aa^	Enzyme-linked immunosorbent assay

^a^Pb: lead.

^b^Cd: cadmium.

^c^Hg: mercury.

^d^Hb: hemoglobin.

^e^RBC: red blood cell.

^f^FVC: forced vital capacity.

^g^FEV_1_: forced expiratory volume in 1 second.

^h^PEF: peak expiratory flow.

^i^ACR: albumin creatinine ratio.

^j^eGFR: estimated glomerular filtration rate.

^k^TC: total cholesterol.

^l^TG: triglyceride.

^m^LDL: low-density lipoprotein.

^n^HDL: high-density lipoprotein.

^o^T_3_: total triiodothyronine.

^p^T_4_: total thyroxine.

^q^TSH: thyroid-stimulating hormone.

^r^ALT: alanine aminotransferase.

^s^ALP: alkaline phosphatase.

^t^AST: aspartate transaminase.

^u^IL-1β: interleukin 1 beta.

^v^IL-6: interleukin-6.

^w^IL-8: interleukin-8.

^x^IP-10: interferon gamma inducible protein-10.

^y^IL-12_P_70: interleukin 12p70.

^z^TNF-α: tumor necrosis factor alpha.

^aa^8-OHdG: 8-hydroxy-2ʹdeoxyguanosine.

### Environmental Sample Collection and Health Risk Assessment

A trained team collected dust and soil samples from e-waste processing sites and nonexposed control sites. To collect the dust samples, the team instructed the workers to collect samples at the end of a workday before cleaning the floor. They swept the floor, desks, and shelves using precleaned paintbrushes and collected the sample into a dustpan. After mixing the samples with a hand trowel, 100 to 150 g of homogenous dust was collected and placed inside an airtight Ziplock plastic bag to prevent access to ambient moisture and other contaminants.

Fieldworkers used a stainless steel spade to clean a 1 m^2^ area, removing visible dust, paper, plastic, or any other organic or inorganic matter. The cleaned surface was divided into 6 equal quadrangles using the hand trowel. Subsequently, the team collected 6 subsamples using a stainless steel spade from the 6 quadrangles. A total of 100 to 150 g of soil was collected from the top surface layer of soil at a 0- to 6-cm depth to get the most precise examination of soil contamination with heavy metals. Samples were labeled, transported, and stored at room temperature at icddr,b for analyses. In addition, the team recorded GPS information at every sampling site. We will send soil and dust samples to the Bangladesh Council of Scientific and Industrial Research to quantify Pb, Cd, Hg, Cr, zinc, copper, selenium, arsenic, nickel, cobalt, beryllium, and vanadium levels using an inductively coupled plasma mass spectrometer (2000; NexION).

To determine the contamination level of both dust and soil samples, we will calculate and compare the geoaccumulation index between exposed and nonexposed sites [38]. Evidence suggests that workers are typically exposed to heavy metals through ingestion, inhalation, and dermal contact. We will calculate the average chronic daily intake through ingestion, inhalation, and skin according to the Exposure Factors Handbook [39]. Furthermore, the standardized formula will estimate the carcinogenic and noncarcinogenic risks of all available risk-posing species.

### Data Management and Quality Assurance

A data management standard operating procedure was developed, in line with the icddr,b data repository system, considering the range and variation in data types. Questionnaires were programmed, and qualified research assistants collected data using handheld computers. The study investigator consulted daily with the research assistants to check data consistency. Data were downloaded from the devices each week and reviewed by the investigator. The statistical team maintains a SQL server database and provides data sets to the investigators in Stata format (.dta). Once data are available to investigators, they will be checked and cleaned for analysis. All data will be stored and periodically updated on icddr,b’s data repository system. Appropriately coded exposure, biological, and environmental data will be periodically shared with the investigators and stored on the server. 

### Statistical Methods

Data will be presented as mean and SD for normal distribution, or median and IQR for nonnormal distribution. Proportions will be calculated for binary and categorical variables, and chi-square tests will examine the distribution differences of categorical variables between 2 groups. We will use Student *t* test (2-tailed) to compare normally distributed data, whereas the Mann-Whitney *U* test will be used for nonnormal or skewed data. Pearson correlations (2-tailed) will be used to test the associations between different normally distributed variables, whereas Spearman correlation test will be used for the nonnormally distributed data to explore the potential risk factors for heavy metal exposure and the relationships between heavy metals and biological parameters. The correlation result will be presented as correlation coefficients (*r*’s) with *P* values.

Linear regression will be used to measure the impact of possible relevant factors on heavy metal exposure, and multiple stepwise regression will be used to investigate the relevant factors contributing to blood Pb and Cd and hair Hg concentrations considering the collinearity of the independent variables. Multivariable regression analyses will be performed to evaluate the associations between blood Pb and Cd or hair Hg concentrations and clinical parameters. All regression models will be adjusted for potential confounders as covariates; these confounding variables will be identified using directed acyclic graphs. Regression diagnostics will be conducted for all models, including examination of fit, influence, and heteroscedasticity. All statistical tests requiring the assumption of normality will be performed on natural logarithmic-transformed concentrations if required. The significance level will be set at α=.05. Data will be analyzed using Stata statistical software (release 13; StataCorp), and figures will be drawn using R software (version 3.5.2 for Windows; R Foundation for Statistical Computing).

### Qualitative Investigation and Analysis

We will conduct a qualitative investigation to understand the overall management system of e-waste in the context of Bangladesh. Since this aim is not testing a hypothesis, we have not conducted a separate sample size calculation for this exploratory qualitative study. We will visit as many respondents as needed to achieve data saturation—the point at which we no longer gain new information [40]. We will conduct interviews with active workers involved in e-waste collection, refurbishment, crude recycling, and disposal. Additionally, we are planning to conduct interviews with key informants, including government officials and stakeholders working on e-waste. We will use a semistructured questionnaire as a data collection tool. All of the qualitative data will be collected by experienced researchers, and they will be trained on the proposed guideline. All the interviews will be recorded using an audio recorder if the participants permit. The research team will also take additional open-ended field notes, including informal discussions and observations. Audio-recorded in-depth interviews and key informant interviews will be transcribed verbatim in the native Bengali language. Data will be analyzed through systematic thematic analysis using the deductive codes generated before data collection, and any inductive codes generated during data analysis. Later, thematic content analysis will be performed to present the result. All the data will be analyzed using ATLAS.ti software (version 5.2; ATLAS.ti Scientific Software Development).

### Ethics Approval

Before interviews and sample collection, eligible adult respondents provided written informed consent in the local language (Bengali). We acquired consent from the guardian and assent from the child if a worker was aged <18 years and enrolled as a study participant. To minimize the risk of breach of confidentiality, every effort was made to conduct the interviews in private. We made every effort to put the subjects at ease during discussions of sensitive questions by using culturally appropriate terminology or euphemisms where possible and by reminding the subjects at the outset that they are free to withdraw from the study at any point. We will develop anonymous data sets without personal identifiers and maintain participants’ privacy by using deidentified data during storage, analysis, and dissemination. This study protocol has been reviewed and approved by the Research Review Committee and Ethical Review Committee of the icddr,b (PR#19057) and The University of Queensland’s Human Behavioral Ethics Committee (2021/HE001648).

## Results

### Participant Characteristics

A total of 199 exposed and 104 nonexposed individuals were successfully enrolled. Demographics for the participants are presented in Table 2. Participants from the exposed group were younger than the nonexposed participants (31 vs 35 years), and most of participants in both groups were male (160/199, 80.4% and 73/104, 70.2%, respectively). No significant differences were found in terms of marital status, family members, and heights of the participants in the 2 groups (*P*=.85, *P*=.77, and *P*=.46, respectively). The exposed group’s weight, hip and waist circumference, and BMI were lower than the nonexposed group’s (*P*=.03, *P*<.001, *P*<.001, and *P*=.04, respectively). There were significant differences and higher consumption rates of smoking and alcohol in exposed participants than nonexposed participants (both *P*<.001).

**Table 2:** Participant characteristics of exposed and nonexposed populations.

Characteristic			Exposed population (N=199), n (%)		Nonexposed population (N=104), n (%)		*P* value	
Age (year), mean (SD)			31 (12)		35 (15)		.01^a^	
Sex, male, n (%)			160 (80.4)		73 (70.2)		.04^b^	
**Education, n (%)**								<.001^b^
	No education	71 (35.7)		28 (26.9)				
	Up to primary school	97 (48.7)		20 (19.2)				
	Above primary school	31 (15.6)		56 (53.8)				
Married, n (%)			143 (71.8)		76 (73.1)		.85^b^	
Self-owned home, n (%)			47 (23.6)		101 (97.1)		<.001^b^	
Family members, mean (SD)			4.66 (2.18)		4.74 (2.04)		.77^a^	
Monthly income (US $), median (IQR)			234 (140-351)		175.52 (117-234)		<.001^c^	
**Household have own, n (%)**								
	Electricity	193 (97)		104 (100)		.07^b^		
	Mobile phone	183 (92)		103 (99)		.01^b^		
	Television	138 (69.3)		72 (69.2)		.98^b^		
	Refrigerator	110 (55.2)		84 (80.8)		<.001^b^		
	Electric fan	186 (93.5)		103 (99)		.02^b^		
	Sewing machine	36 (18.1)		33 (31.7)		.01^b^		
**Occupation, n (%)**								<.001^b^
	e-Waste workers	199 (100)		N/A^d^				
	Farmer	N/A		31 (30)				
	Small business	N/A		20 (19.2)				
	Student	N/A		18 (17.3)				
	Housewife	N/A		15 (14.4)				
	School teacher	N/A		5 (4.8)				
	Others (service, day labor, tailor, and mason)	N/A		15 (14.4)				
Smoker, n (%)			122 (61.3)		31 (29.8)		<.001^b^	
Alcohol consumption, n (%)			37 (18.6)		3 (2.9)		<.001^b^	
Height (cm), median (IQR)			157.85 (153.03-163.23)		159.03 (152.53-165.43)		.46^c^	
Weight (kg), (median (IQR)			51.9 (44.77-61.1)		55.5 (48.68-64.97)		.03^c^	
BMI (kg/m^2^), median (IQR)			20.4 (18-24.4)		21.87 (19.25-25.5)		.04^c^	
Hip circumference (cm), median (IQR)			33.45 (31.2-35.95)		36.5 (32.77-82.30)		<.001^c^	
Waist circumference (cm), median (IQR)			29.35 (25.85-33.75)		33.8 (27.62-74.55)		<.001^c^	

^a^Independent-sample *t* test.

^b^Chi-square test.

^c^Mann-Whitney *U* test.

^d^N/A: not applicable.

### Dissemination

We will share the study findings with the Department of Environment of the government of Bangladesh and other partner nongovernmental organizations working on environmental issues to ensure a safe environment. We will discuss the scope and limitations of e-waste management based on our study findings. We will develop technical and policy-level interventions and capacity building and increase public awareness of these environmental hazards. We will develop abstracts for international conferences to international audience for dissemination. We will develop manuscripts and submit them to peer-reviewed journals.

## Discussion

To the best of our knowledge, this will be the first epidemiological study to assess the health effects of e-waste exposure among current e-waste workers in Bangladesh. This study has several strengths. First, we collect extensive data on potential exposures, covariates, and outcomes that will help determine valid and meaningful associations between different exposure-outcome combinations. We collected biological samples (blood, urine, and hair) from which a comprehensive health assessment can be measured. We also collected environmental samples that allows us to determine the correlation between the concentrations of environmental heavy metals and levels in participants’ biological specimens. Second, most of the outcomes planned in this study are objective measures that will increase the validity of our findings and ease the risk of reporting bias.

There are some limitations as well. Study subjects may have been exposed to toxicants other than those we have measured, such as other heavy metals and persistent organic pollutants, and these exposures may be correlated with those we have measured. As such, attributing causality to associations between Pb, Cd, and Hg and adverse health outcomes may be problematic. Moreover, we encountered several obstacles during data collection. First, the COVID-19 surge delayed our data collection process, and we had to halt the data collection on multiple occasions to comply with COVID-19 guidelines. Second, workers had limited work scope during this critical time, and many of them left their job, which may have reduced the continuous exposure. Additionally, there is a possibility of information bias and a possible overreporting of symptoms, especially respiratory and female reproductive issues among the exposed individuals.

Despite these limitations, the danger to the population involved in e-waste handling is largely undocumented, and this study will provide important information. The research findings could provide data to support decision-making and the formation of action plans following the Bangladesh e-waste management road map.

## Appendix

Multimedia Appendix 1 Peer-review report by the International Centre for Diarrhoeal Disease Research, Bangladesh (icddr,b) - Research Review Committee (Dhaka, Bangladesh).

## Abbreviations

Cd: cadmium
Cr: chromium
Hg: mercury
icddr,b: International Centre for Diarrheal Disease Research, Bangladesh
Pb: lead
