# Supplementary material for: Ecological Burden of e-Waste in Bangladesh—an Assessment to Measure the Exposure to e-Waste and Associated Health Outcomes: Protocol for a Cross-sectional Study
Source: JMIR Res Protoc. 2022 Aug 16;11(8):e38201. doi: 10.2196/38201 (PMC9428780; doi:10.2196/38201)
Supplement: Multimedia Appendix 1 [file resprot_v11i8e38201_app1.pdf]

icdr,b

Memorandum

08 July 2019

To: Mr Sarker Masud Parvez
Principal Investigator of research protocol # PR-19057
Infectious Diseases Division (IDD)

From: Shafiqul Alam Sarker, MD, PhD, FRCP
Chairperson
Research Review Committee (RRC)

Sub: Research protocol # PR-19057

Thank you for submitting your research protocol # PR-19057 titled "Characterizing the Environmental Burden of E-waste and their Impact on Children and Women Health" for consideration of the RRC and present it before the Committee in its special meeting held on 04 July 2019. This is to inform you that after review and discussion, the committee made the following observations on the protocol:

a) Proper spacing between two words and word to reference should be reviewed throughout the protocol: For example - Page 1, 15, 16, 18 etc.

b) Lots of typos and spelling mistakes. So, spelling should be reviewed throughout the protocol.

c) Inappropriate article and punctuation use should be reviewed throughout the protocol. For example, indefinite article missing before exponential, opportunity, and vulnerable; definite article missing before human and outcome on page 13.

d) Is it possible to mention the other sites in Bangladesh where the project work will be implemented? (Page 6, under the title of "Project/Study Site" Savar & Others...)

e) Please answer to the question whichever is appropriate, (page 9) Dissemination Type: Sharing with international agency-Yes/No. Either one should be checked.

f) Confirm the abbreviation such as PBDE instead of PDBE, CDC= Center for Disease Control and Prevention instead of Center for Disease Control, MMT= Million metric ton.

g) Why the study is not going to add health impact screening of men worker along with child and women health is not clearly stated.

h) Does the study have any negative control of E-waste health impact using people from no exposure area?

i) Is this data from Bangladesh or globally generated E-Waste amount? Mention it, Page 15, Line 16-17 (The amount of e-waste generated in

Postal address
icddr,b
GPO Box 128
Dhaka 1000
Bangladesh

Delivery & visitors
icddr,b
Mohakhali
Dhaka 1212
Bangladesh

T (88 02) 9827001-10
F (88 02) 9827075, 9827077
E info@icddrb.org
www.icddrb.org

2016 was 44.7 million metric tons (MMT), an 8% increase from 41.8 MMT in 2014, and is projected to reach 52.2 MMT by 2021).

j) Suggested to use the word "measure" instead of enumerate for lead and mercury. Page 18, Line 9 under the title of Objective 2 (measure the severity of BLLs and BCLs).

k) Text alignment is suggested.

l) Signature of Co-Investigator, Dr Nafisa Islam is missing.

m) What type of soil contamination are the investigators expected? Are they going to measure silicon? How different will it be from other industrial studies? Are the types of contaminations expected to be different?

n) Is there any dumping happening in Burigonga also? Are investigators going to collect samples from Burigonga?

o) The level of lead is very much associated with stunting. There is a publication in this regard and investigators can use that article. The investigators might go to measure the renal functions also.

p) Anthropometry measurement of participants may be included in the study.

q) Define nutritional implications? Will nutritional status be estimated? Needs clarification.

r) Do the investigators have any control area of the study? It is important to have a control arm.

s) PABX numbers with extensions should be deleted from the consent forms. Provided mobile number and direct phone number is alright.

You are, therefore, advised to address each of the above mentioned observations of the committee and submit the revised version of the protocol for consideration by the chair.

Thank you once again.

Cc: Senior Director, IDD
